# Soluble CD44 Interacts with Intermediate Filament Protein Vimentin on Endothelial Cell Surface

**DOI:** 10.1371/journal.pone.0029305

**Published:** 2011-12-21

**Authors:** Taavi Päll, Anne Pink, Lagle Kasak, Marina Turkina, Wally Anderson, Andres Valkna, Priit Kogerman

**Affiliations:** 1 Department of Gene Technology, Tallinn University of Technology, Tallinn, Estonia; 2 Competence Center for Cancer Research, Tallinn, Estonia; Université Joseph Fourier, France

## Abstract

CD44 is a cell surface glycoprotein that functions as hyaluronan receptor. Mouse and human serum contain substantial amounts of soluble CD44, generated either by shedding or alternative splicing. During inflammation and in cancer patients serum levels of soluble CD44 are significantly increased. Experimentally, soluble CD44 overexpression blocks cancer cell adhesion to HA. We have previously found that recombinant CD44 hyaluronan binding domain (CD44HABD) and its non-HA-binding mutant inhibited tumor xenograft growth, angiogenesis, and endothelial cell proliferation. These data suggested an additional target other than HA for CD44HABD. By using non-HA-binding CD44HABD Arg41Ala, Arg78Ser, and Tyr79Ser-triple mutant (CD443MUT) we have identified intermediate filament protein vimentin as a novel interaction partner of CD44. We found that vimentin is expressed on the cell surface of human umbilical vein endothelial cells (HUVEC). Endogenous CD44 and vimentin coprecipitate from HUVECs, and when overexpressed in vimentin-negative MCF-7 cells. By using deletion mutants, we found that CD44HABD and CD443MUT bind vimentin N-terminal head domain. CD443MUT binds vimentin in solution with a Kd in range of 12–37 nM, and immobilised vimentin with Kd of 74 nM. CD443MUT binds to HUVEC and recombinant vimentin displaces CD443MUT from its binding sites. CD44HABD and CD443MUT were internalized by wild-type endothelial cells, but not by lung endothelial cells isolated from vimentin knock-out mice. Together, these data suggest that vimentin provides a specific binding site for soluble CD44 on endothelial cells.

## Introduction

CD44 transmembrane glycoprotein functions as hyaluronan (HA) receptor. CD44 has functions in a lymphocyte homing, mediates cell adhesion to HA and HA metabolism. CD44 is expressed on many cell types including endothelial cells (EC) and has multiple alternatively spliced isoforms. CD44 plays a significant role in tumor malignancy. High levels of CD44 expression on tumor cells is sufficient to establish metastatic behavior [1], [2]. CD44 is involved in pathological angiogenesis, as its expression is elevated in tumor vasculature, and CD44 expression can be induced in cultured ECs by angiogenic growth factors [3] Furthermore, CD44 knockout mice show reduced vascularisation of tumor xenografts and Matrigel plugs [4]. In addition to cell surface expression, CD44 is present in soluble form in lymph and serum [5] or bound to extracellular matrix [6]. Soluble CD44 is generated either by alternative splicing [7] or, more importantly, by ectodomain shedding by matrix metalloproteases [8], [9].The size of shed CD44 is highly heterogeneous because of glycosylations and variant exons [5], [9]–[11]. The serum concentration of sCD44 in mice is known to range between 490 to 2100 ng/ml [5]. Studies of sCD44 in the sera of non-Hodgkin's lymphoma and breast cancer patients show that physiological sCD44 level in healthy persons is in the range of 250 to 500 ng/ml [12]–[14]. The serum concentration of sCD44 in healthy individuals is ∼3 nM whereas it was shown to be significantly elevated in patients with advanced gastric (24 nM) or colon cancer (31 nM) [11]. Elevated serum sCD44 or sCD44v6 is a predictor of poor therapeutic outcome in non-Hodgkin's lymphoma or breast cancer patients, respectively [12], [15].The source of sCD44 are lymphocytes, macrophages, ECs, and tumor cells [10], [11], [16]. In non-Hodgkin's lymphoma, the source of elevated sCD44 are lymphoma cells, and sCD44 levels decrease after treatment in patients with complete remission [10], [17]. Endothelial and macrophage CD44 expression is increased in atheromas and CD44 shedding from EC and macrophages is stimulated by proinflammatory cytokines [16].

Tumors are surrounded by HA-rich ECM. When overexpressed in tumor cells, soluble CD44 can function as an antagonist to cell membrane CD44 and block its binding to ECM HA. Overexpression of soluble forms of CD44 inhibits HA-adhesion of mouse mammary carcinoma or melanoma cells and caused inhibition of tumor cell proliferation, and reduced tumorigenicity [18]–[20]. CD44 knockout in mouse breast cancer model caused increased numbers of lung metastases, which correlated with reduced invasion of CD44-expressing metastatic breast cancer cell lines into HA-containing collagen matrixes [21].

CD44 binds HA via the link module in its N-terminal domain. The link module is approximately 100 amino acids long and consists of two alpha helices and two triple-stranded antiparallel beta sheets, stabilized by two disulphide bridges [22]. The structure of CD44 HABD has an additional lobe consisting of four beta strands formed by the residues flanking the core link module [23], [24]. This enlarged structure is stabilized by an additional disulphide bridge between flanking regions. Together, the human CD44 HABD structure consists amino acids 21–169. The HA-binding surface of CD44 is exclusively covered by the link module and its flanking regions do not contribute to the HA binding [23]. The critical residues in CD44 HA-binding surface directly involved in binding are Arg41, Tyr42, Arg78, and Tyr79, according to studies of human CD44 [23], [25]. Glycosylation of Asn25 and Asn125 within CD44 HABD is involved in regulation of HA binding [26]. Altogether, CD44 has five N-glycosylation sites (Asn25, Asn57, Asn100, Asn110, Asn120) within its HABD. Bacterially expressed recombinant human CD44 HABD containing amino acids 20–178 binds HA comparably to glycosylated CD44-Rg fusion protein [24]. HA binding function is also retained by a recombinant human CD44HABD containing amino acids 21–132, whereas HA binding was abolished by the mutations in Arg41, Arg78, and Tyr79 [27].

Vimentin intermediate filaments comprises supporting framework within cells. Vimentin functions in intracellular vesicular transport, including β1-integrin trafficking [28], transport of lysosomal membrane proteins by binding AP-3 complex [29], and as a cytosolic reservoir for tSNARE SNAP23 [30]. Importantly, vimentin knockout cells apparently retain intact receptor-mediated endocytosis, as transferrin receptor level and distribution is normal [29], [30]. Vimentin-deficient mice reproduce and develop normally [31], however, they show reduced elasticity of arteries, decreased nitric oxide production and elevated endothelin [32], [33]. Vimentin is expressed on cell surface in several cell types, including TNF-α induced macrophages [34], cutaneous T-cell lymphoma [35], platelets [36], and brain microvascular endothelial cells [37]. Vimentin extracellular ligands include vitronectin/PAI-1 complex [36], and *E. coli* IbeA protein [37]. Vimentin is a antiangiogenesis target overexpressed on tumor endothelium *in vivo*. Anti-vimentin antibody treatment inhibited subcutaneous tumor xenograft growth and tumor blood vessel density in mice, suggesting that vimentin is localized to the cell surface in tumor endothelial cells [38].

CD44 and vimentin are both detectable from membrane lipid raft fractions [39]–[41] and from clathrin-independent pathway endocytic vesicles in fibroblasts [42]. CD44 and vimentin are upregulated during epithelial-mesenchymal transition (EMT) of cancer cells. Mammary epithelial cells undergoing EMT downregulate epithelial genes and upregulate mesenchymal genes, such as E-cadherin, N-cadherin and vimentin, respectively. Suppression of standard CD44 isoform in Snail- or TGF-β-induced human mammary epithelial cells inhibits EMT, accompanied by vimentin downregulation [43].

We have previously found that recombinant CD44 HABD 21–132, as a model for soluble CD44, inhibited human subcutaneous tumor xenograft growth in mice, angiogenesis in chick chorio-allantoic membrane, and EC proliferation [27]. Surprisingly, these CD44HABD functions were independent of its HA-binding propery, as non-HA-binding mutant was similarly effective. Therefore, we proposed that CD44HABD could bind additionally to a different ligand than HA. In this study, we used CD44HABD non-HA-binding mutant as a bait in GST pull-down assay and identified vimentin as a novel CD44 interacting protein.

## Results

### Identification of vimentin as CD44 HABD-binding protein

**Figure 1:**
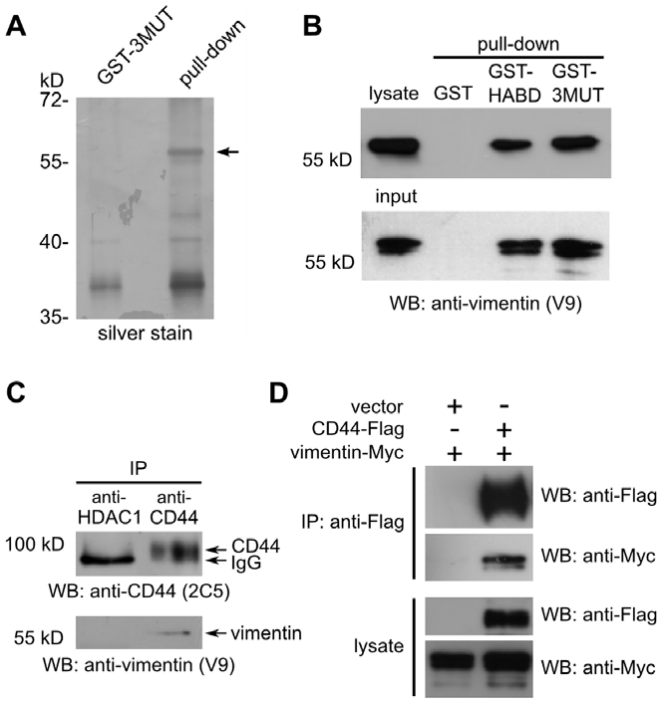
Identification of vimentin as CD44HABD-binding protein. (A) HUVEC lysate was used in GST pull-down to identify CD443MUT interacting proteins. Lysate was incubated with GST-CD443MUT (GST-3MUT) coated beads. Bound proteins were eluted using reduced glutathione and analyzed by SDS-PAGE and silver staining. GST-3MUT precipitated protein band (shown by arrow) was cut off from gel, trypsinolyzed and analyzed by MALDI-TOF MS. This protein was identified as vimentin. (B, upper panel) Vimentin pull-down by CD44HABD (GST-HABD) and GST-3MUT was confirmed by immunoblotting using anti-vimentin V9 antibody. (B, lower panel) GST-HABD and GST-3MUT pull-down recombinant vimentin. (C) Coimmunoprecipitation of vimentin with CD44 from HUVEC lysate. Anti-HDAC-1 antibody was used as a negative control (see Materials and methods). (D) Coimmunoprecipitation of over-expressed vimentin-Myc with CD44-Flag from MCF-7 lysates using tag-specific antibodies.

To identify EC target of CD44 HABD 21–132 (CD44HABD) and its non-HA-binding mutant CD44HABD^R41AR78SY79S^ (CD443MUT), we used GST pull-down from HUVEC lysate. Silver staining of pull-down reactions separated by SDS-PAGE revealed that GST-tagged CD443MUT precipitated a 60 kD protein (Figure 1A). This protein was identified by MALDI-TOF-MS protein fingerprinting as vimentin. To confirm that CD44HABD-proteins pull down vimentin, we used anti-vimentin (V9) immunoblotting. Immunoblotting confirmed that GST-tagged CD44HABD and CD443MUT pulled down endogenous vimentin from HUVEC lysates (Figure 1B, upper panel). To determine whether CD44HABD and CD443MUT bind vimentin directly, we used recombinant vimentin in the GST pull-down assay. We found that both CD44HABD and CD443MUT were able to pull down recombinant vimentin, suggesting that CD44 interacts with vimentin directly (Figure 1B, lower panel). We next used immunoprecipitation (IP) to determine whether endogenous CD44 and vimentin associate in EC. HUVEC lysate was immunoprecipitated using anti-CD44 (MEM-263) antibody and immunoprecipitates were subsequently analyzed by immunoblotting. We found that a minor population of vimentin coimmunoprecipitated with CD44 from HUVEC lysate (Figure 1C). We also tested whether anti-vimentin antibodies coimmunoprecipitate CD44. However, we were not able to detect CD44 from anti-vimentin IPs (A.P., unpublished data). To further confirm full-length CD44 and vimentin association we overexpressed C-terminally Flag-tagged CD44 standard isoform and Myc-tagged vimentin in vimentin nonexpressing MCF-7 cells. Overexpressed vimentin was exposed to the cell surface as detected by cell surface biotinylation (Figure S1). Immunoprecipitation results showed that anti-Flag immunoprecipitated a vimentin-Myc from CD44-Flag transfected cells (Figure 1D).

### Vimentin and CD443MUT in vitro binding affinity

CD443MUT interaction with recombinant full length human vimentin was further characterized by isothermal titration calorimetry (ITC) and by surface plasmon resonance (SPR). We used two different preparations of CD443MUT. ITC experiments showed that CD443MUT binds to recombinant vimentin with Kd in 12–37 nM range with stoichiometry (vimentin/CD443MUT) of ≈7 mol/mol (Table 1). SPR experiments were carried out with vimentin immobilized into measuring cell. Kinetic analysis by SPR revealed that binding of CD443MUT to immobilized vimentin is described by a two-site ligand binding model. CD443MUT bound to a high-affinity site of immobilized vimentin with Kd 74 nM and Kd for low affinity site was 15 µM (Table 2). Analysis of kinetic data using equilibrium response values resulted in 15±2 µM Kd. The stoichiometry of vimentin/CD443MUT complex in SPR experiment was measured≈6 mol/mol .

**Table 1:** Summary of Kd values for CD443MUT and vimentin interaction measured by ITC.

CD443MUT preparation	CD443MUT (µM)	Vimentin (µM)	Kd (M)	n^a^ (mol/mol)
A	4.2	1.8	1.2·10−8±10−9	9.9±0.5
	1.5	0.5	3.7·10−8±10−9	
B	4.2	1.8	1.8·10−8±10−9	7.2±0.3
	0.9	0.5	2.3·10−8±10−9	

a , stoichiometry (vimentin/CD443MUT).

**Table 2:** Kinetic parameters for binding of CD443MUT to vimentin measured by SPR.

Kass1 (M^−1^ s^−1^)×10^3^	Kass2 (M^−1^ s^−1^)	Kdiss1 (s^−1^)×10^−4^	Kdiss2 (s^−1^)×10^−3^	Kd (µM) Kdiss1/Kass1	Kd (µM) equation 1	*n* (mol/mol)
7.6±0.1	183±7	5.6±0.1	1.9±0.1	0.074	15±2	6.2

### Mapping of vimentin CD44-binding region

**Figure 2:**
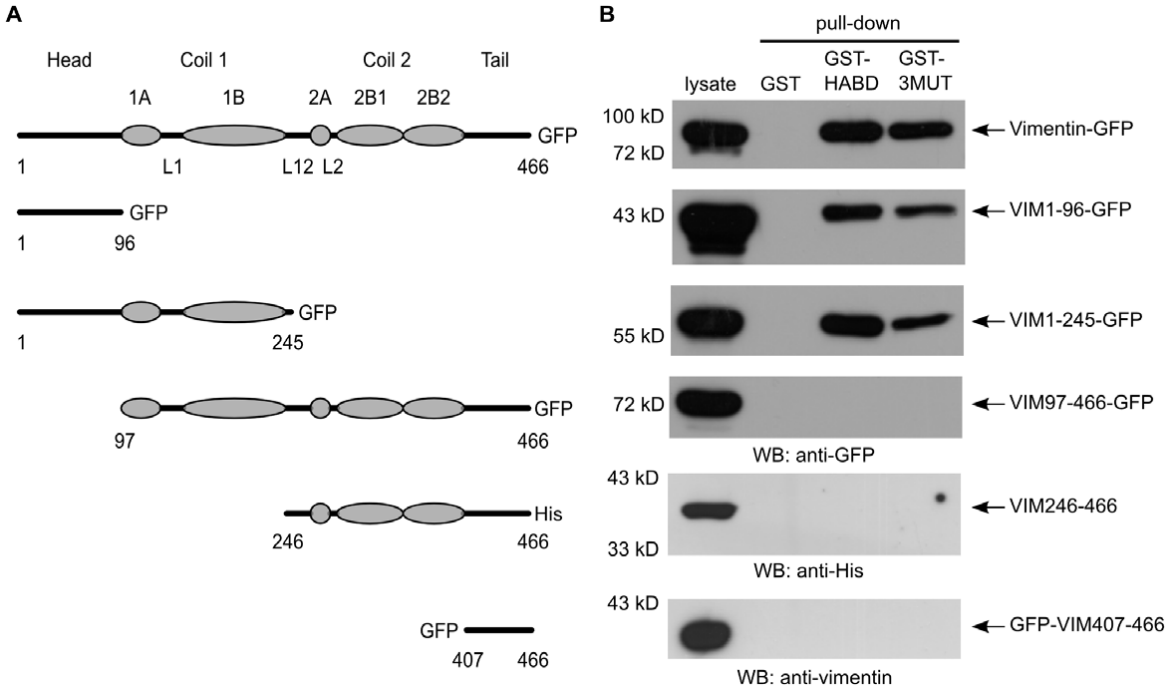
CD443MUT binds vimentin N-terminal head domain. (A) A diagram of vimentin sub-domains and deletion mutants used in pull-down reactions. Ellipses represent alpha-helices in coiled-coil domains and L1-L2 mark linker regions. GFP, green fluorescent protein. (B) GST pull-down reactions were performed from cell lysates transfected with full length vimentin or its deletion mutants (see Materials and methods). Eluates from pull-downs were analyzed by immunoblotting.

To map CD44-binding region in vimentin, we generated truncated vimentin constructs (Figure 2A). Vimentin deletion mutant VIM1-96 contains only head domain, VIM1-245 contains head domain and alpha-helices 1A-B, and VIM97-466 mutant lacks the head domain (aa numbering according to human vimentin). VIM246-466 mutant contains C-terminal half of the protein starting from alpha-helices 2A-B. VIM407-466 contains the tail domain. Lysates of MCF-7 cells, expressing either full-length vimentin or its deletion mutants, were used in GST pull-down with CD44HABD or CD443MUT. Pull-downs were analyzed by immunoblotting using tag-specific antibodies. This analysis showed that CD44HABD and CD443MUT bound only vimentin deletion mutants containing the head domain (VIM1-96 and VIM1-245; Figure 2B). Deletion of the head domain was sufficient to abolish binding of vimentin to CD44HABD and CD443MUT(VIM97-477, VIM246-466 or VIM407-466).

### Cell surface vimentin and CD443MUT-vimentin interaction is induced by VEGF

**Figure 3:**
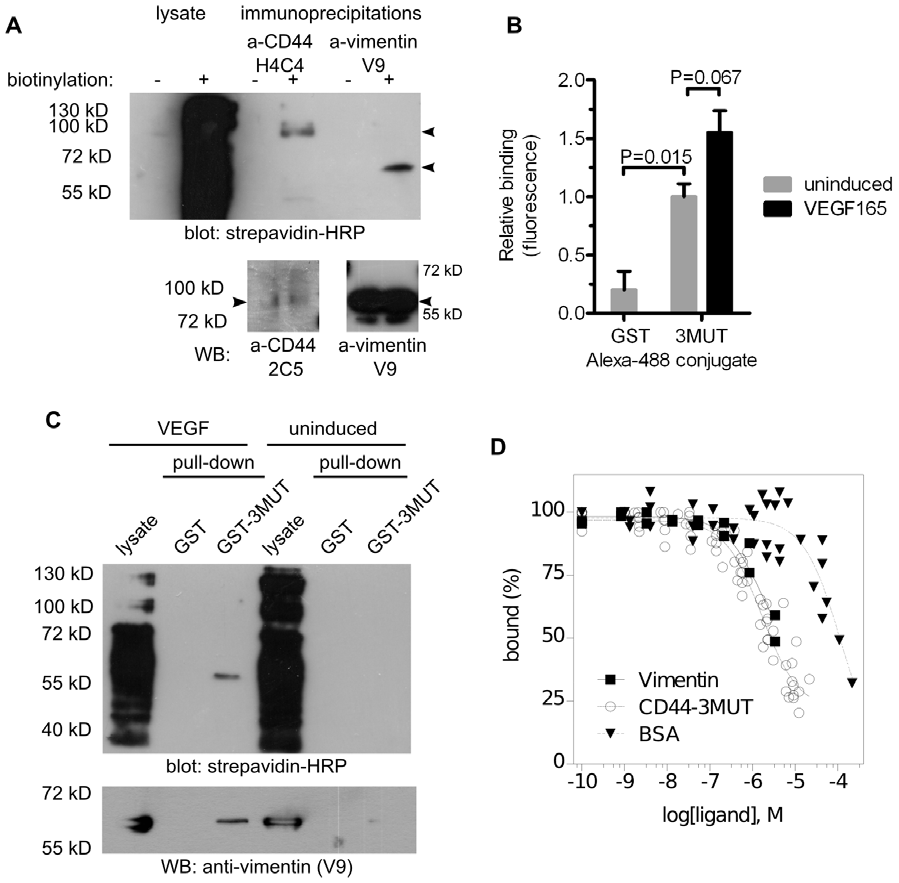
VEGF induces cell surface vimentin and CD443MUT cellular binding. (A) For detection of cell surface vimentin, asynchronously growing live adherent HUVEC were cell surface biotinylated and lysate was used for immunoprecipitation using anti-vimentin or anti-CD44 antibodies. Immunoprecipitated proteins were detected by immunoblotting using strepavidin-HRP (upper panel) or specific antibodies (lower panels). (B) 6 hour serum-starved HUVEC were induced for 30 min with VEGF165, followed by incubation on ice with Alexa Fluor 488-labeled CD443MUT (3MUT). GST Alexa Fluor 488 conjugate was used as negative control. Cellular binding of A488-conjugated proteins was analyzed by FACS. Bars represent average geomean of fluorescence from three experiments (mean ± SE). (C) Overnight serum-starved HUVEC were induced for 1 hour with VEGF165, followed by cell surface biotinylation. Lysate from biotinylated cells was used in pull-down using GST-3MUT. Precipitated proteins were detected by immunoblotting using strepavidin-HRP (upper panel) or anti-vimentin antibody (lower panel). (D) For displacement assay, cells were resuspended in incubation buffer in 96-well plate. CD443MUT, vimentin or BSA at different concentrations was added to the wells along with ^125^I-labeled CD443MUT. Reactions were incubated overnight at 4°C. After incubation, reactions were stopped by filtration through glass fiber filters blocked with BSA. Filters were washed with PBS and bound radioactivity was measured using gamma counter. The curves represent global fitting of normalized radioligand binding data from two to nine experiments.

To detect cell surface vimentin, we performed biotinylation of cell surface proteins of adherent living HUVEC, followed by IP of vimentin from cell lysates. Biotinylated proteins were detected by immunoblotting using HRP-conjugated strepavidin. We found that anti-vimentin (V9) antibody immunoprecipitated from HUVEC lysate a 60 kD biotinylated protein. We used anti-CD44 (H4C4) antibody as positive control and found that it IPd a 100 kD biotinylated protein. These proteins correspond to expected sizes of vimentin and endothelial CD44, respectively (Figure 3A upper panel). The identity of biotinylated proteins was confirmed by immunoblotting with vimentin- or CD44-specific antibodies (Figure 3A lower panel).

Next, we decided to test whether CD443MUT cellular binding can be induced with angiogenic growth factors. To determine the effect of angiogenic stimulus on CD443MUT cellular binding we induced 6 h serum starved HUVEC 30 min with VEGF165 at 37°C. Then we incubated cells with Alexa Fluor 488-labeled CD443MUT at 4°C. CD443MUT-A488 cellular binding was quantitated using flow cytometry. We found a significant binding of CD443MUT-A488 to HUVEC compared to GST-A488 control (P = 0.015, n = 3, unpaired t-test). Under these conditions ∼20% cells bound CD443MUT. VEGF treatment induced a further increase in CD443MUT cellular binding compared to non-induced cells, although this result was statistically marginally significant (P = 0.067, n = 3; Figure 3B). To confirm that VEGF induces cell surface vimentin binding sites for CD443MUT, we used cell surface biotinylation of HUVEC followed by GST pull-down with CD443MUT. For this, overnight serum starved HUVEC were induced 1 hour with VEGF or left non-induced, followed by cell surface biotinylation of live adherent cells. GST-CD443MUT or GST alone were used in pull-downs from cell-surface biotinylated HUVEC lysates. Subsecuently, precipitated proteins were detected by western blotting either by strepavidin-HRP or anti-vimentin (V9) antibody. We found that CD443MUT pulled down a 60 kD biotinylated protein from VEGF-stimulated but not from serum starved cells. This protein turned out to be vimentin since it could be detected with a vimentin-specific antibody (Figure 3C).

### Vimentin displaces CD443MUT from HUVEC

To further characterize CD443MUT and vimentin interaction on HUVECs we measured the ability of vimentin to compete with ^125^I-labeled CD443MUT for cellular binding. The results of displacement binding experiments showed that CD443MUT displaced itself from HUVEC with logEC50 −5.8±0.05 M (EC50 = 1.57 µM, n = 9; Figure 3D). Vimentin displaced CD443MUT from HUVEC with logEC50 −5.37±0.21 M (EC50 = 4.26 µM , n = 2) which is not significantly different from displacement by CD443MUT itself (extra sum of squares F-test, *P* = 0.0711; F = 3.298 (1,171)). BSA did not displace CD443MUT effectively, with logEC −3.93±0.06 M (EC50 = 117 µM, n = 4).

### CD44HABD endocytosis by HUVEC

**Figure 4:**
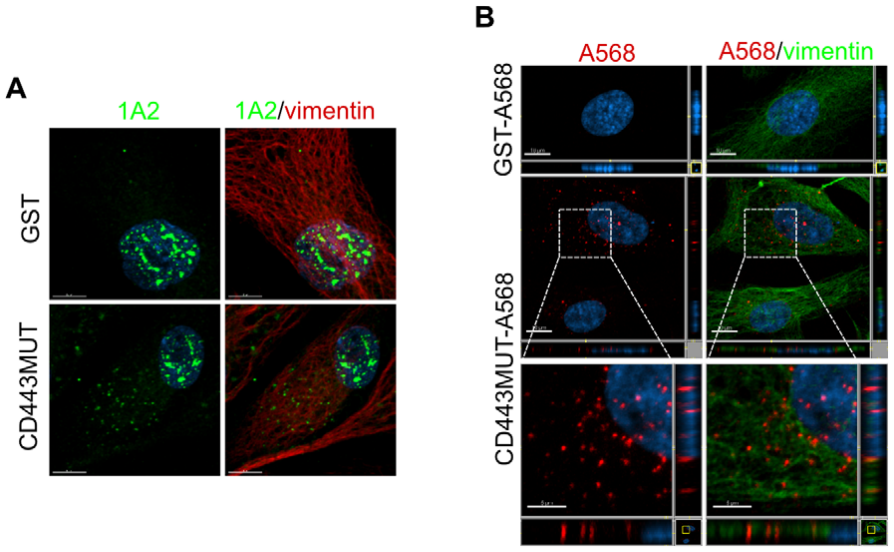
CD443MUT endocytosis by HUVEC. HUVEC were grown overnight on glass slides and incubated for 30 min at 37°C with 1 µM unlabeled or Alexa Fluor 568-labeled CD443MUT or GST. Cells were analyzed by confocal microscopy. (A) Uptake of unlabeled CD443MUT by HUVEC was detected with anti-CD443MUT mouse mAb 1A2 (green). Vimentin intermediate filaments were detected with rabbit polyclonal antibody (red). Nuclei were stained with Hoechst (blue). Images are maximum intensity projections, generated along the z-axis of image stack. Scale bars, 10 µm. (B) Internalization of directly Alexa Fluor 568-labeled CD443MUT by HUVEC (red). Vimentin (green) was detected with V9 mAb. Scale bars, upper and middle panels 10 µm; insets 5 µm.

Given that vimentin provides specific binding site for CD443MUT on EC, we decided to test whether CD443MUT is endocytosed upon binding to cell surface vimentin. We incubated HUVEC with unlabeled CD443MUT for 30 min at 37°C to allow internalization. CD443MUT was detected by immunofluorescence confocal microscopy using CD443MUT specific mouse monoclonal antibody 1A2 (Figure S2). Recombinant GST uptake was used as a control. The results showed that CD443MUT was readily endocytosed by HUVEC and displayed a vesicular localization pattern (Figure 4A). Next, we used CD443MUT directly conjugated to Alexa Fluor 568 for internalization assay. CD443MUT-A568 was endocytosed and distributed in HUVEC cytoplasm similarly to unlabeled CD443MUT (Figure 4B). HUVECs express vimentin at high level, and endocytosed CD443MUT-containing vesicles were surrounded by a dense network of vimentin intermediate filaments, however, there was no direct colocalization of CD443MUT with vimentin filaments (Figure 4A and B).

**Figure 5:**
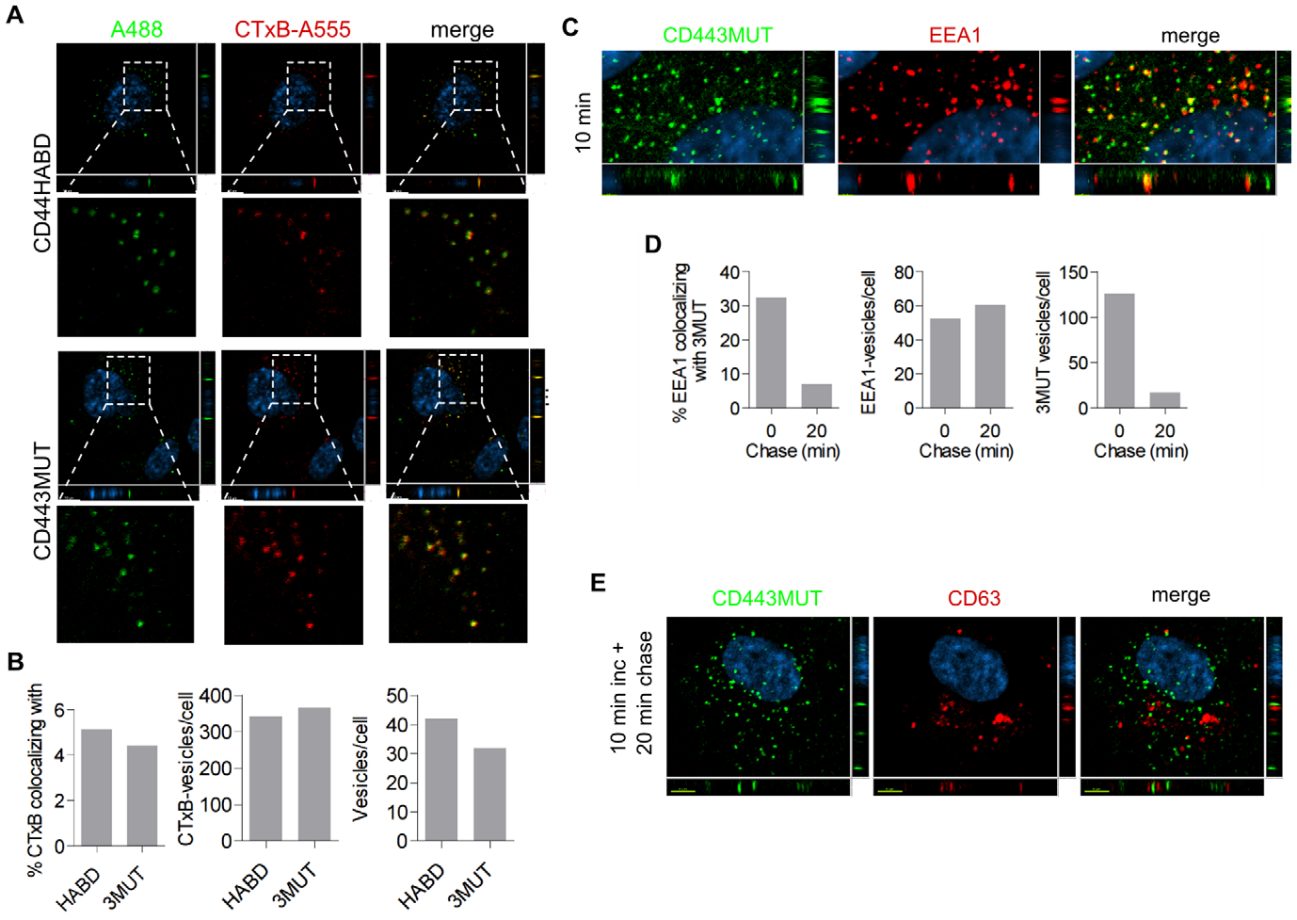
Analysis of endocytosed CD443MUT localization. (A) HUVEC were incubated with A488-labeled CD44HABD or CD443MUT (green) in the presence of CTxB-A555 (red) for 30 min. Nuclei were stained with Hoechst. Images show single confocal plane. Scale bars, 10 µm. (B) Colocalization analysis of CD44HABD (HABD) and CD443MUT (3MUT) with CTxB. Left, the fraction of CTxB-vesicles colocalizing with HABD (n = 39 cells) or 3MUT (n = 38 cells). Middle, the number of CTxB-vesicles per cell; right, the number of HABD- or 3MUT-containing vesicles per cell. (C–E) HUVEC were incubated with CD443MUT-A488 for 10 min after which CD443MUT-containing media was changed to 10% FBS HUVEC growth media and cells were further incubated for 20 min. Then cells were fixed and stained with anti-EEA1 or anti-CD63 antibodies. (C) Localization of 3MUT- and early endosomal marker EEA1-positive veicles after 10 min incubation in HUVEC. (D) Quantitation of EEA1-vesicles colocalizing with CD443MUT after 10 min incubation (n = 26 cells) and after 20 min chase (n = 40 cells; left). The number of EEA1- and 3MUT vesicles per cell (middle and left, respectively). (E) Localization of internalized 3MUT and late endosomal protein CD63-positive vesicles. Scale bars, 2 µm (C) and 5 µm (E).

Next, we used a generic endocytosis marker cholera toxin B conjugated to Alexa Fluor 555 (CTxB-A555) to trace CD443MUT following endocytosis. We found that after 30 min uptake Alexa Fluor 488-labeled CD44HABD as well as -3MUT colocalized with CTxB-A555 positive structures (Figure 5A). We quantitated colocalization of CTxB with CD44HABD and CD443MUT from single slices of confocal image stacks as described in Materials and Methods. Altogether, ∼2.6·10^4^ CTxB-positive vesicles were analyzed from CD44HABD- (*n* = 39) or CD443MUT-incubated cells (*n* = 38). As shown in Figure 5B, approximately 4–5% of CTxB-vesicles colocalized and showed positive correlation with CD44HABD (average Pearson's r = 0.469, 95% CI 0.438 to 0.498, df = 679, *P*<0.0001) or CD443MUT (r = 0.532, 95% CI 0.503 to 0.531, df = 608, *P*<0.0001). We next analyzed CD443MUT-A488 colocalization with early endosome marker EEA1 in HUVEC after 10 min uptake followed by 20 min chase. We found that CD443MUT-A488 showed extensive colocalization with EEA1-positive vesicles after 10 min incubation (Figure 5C). Quantitation of CD443MUT and EEA1 colocalization in ∼6.5·10^3^ EEA1-endosomes showed that 32% of EEA1-endosomes colocalized with CD443MUT after 10 min incubation (r = 0.311, 95% CI 0.266 to 0.355, df = 407, *P*<0.0001), whereas a fraction of EEA1-endosomes showing colocalization falled to 7% after 20 min chase (r = 0.321, 95% CI 0.251 to 0.388, df = 172, *P*<0.0001) following the incubation (Figure 5D). The number of CD443MUT-vesicles in cells reduced during 20 min chase by ∼7.5 times (Figure 5D, rightmost panel) suggesting trafficking of CD44 to late endosomal-lysosomal degradation pathway. Therefore, we next analyzed whether CD443MUT is targeted to the CD63-positive late endosomal compartment after 20 min chase following a 10 min pulse with CD443MUT-A488. However, we found that CD443MUT-A488 showed no significant accumulation within anti-CD63 staining vesicles after 20 min (Figure 5E) or 50 min chase (data not shown). Together, these results indicate that recombinant CD44HABD and CD443MUT are endocytosed and reach early endosomal compartment.

### CD443MUT endocytosis is inhibited in ECs derived from vimentin-null mice

**Figure 6:**
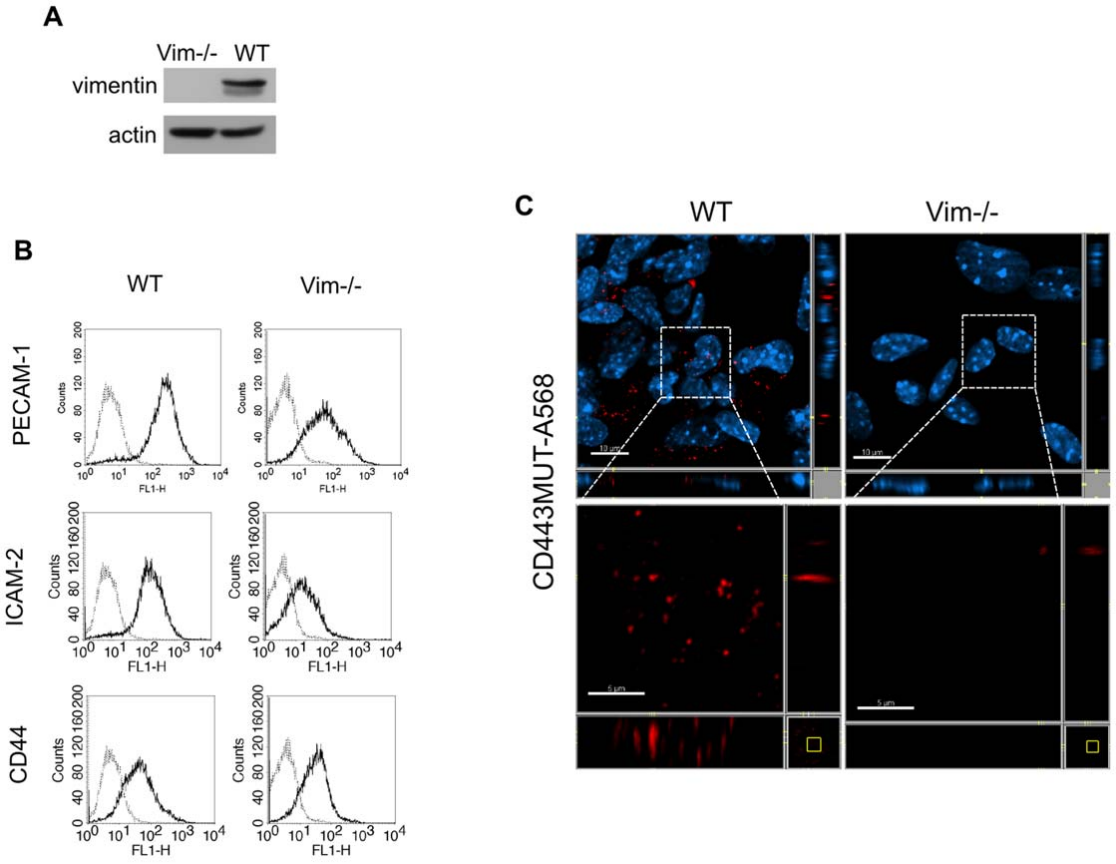
Vimentin dependent endocytosis of CD443MUT. MLEC were isolated either from wild-type (WT) or vimentin-null mice. (A) Immunoblot of WT of Vim−/− MLEC lysates with anti-vimentin rabbit polyclonal antibody. (B) FACS analysis of MLEC for cell surface markers with either anti-PECAM-1, anti-ICAM-2 or anti-CD44 antibodies (black lines). Gray lines, no primary antibody controls. (C) MLEC-s were incubated with CD443MUT-A568 (red) for 30 min and processed for immunofluorescence. Scale bars, upper panels 10 µm; insets 5 µm.

To test directly whether vimentin mediates CD443MUT internalization, we isolated lung endothelial cells from wild-type (WT) or vimentin-null mice (Figure 6A). We characterized isolated mouse lung endothelial cells (MLEC) for endothelial-specific cell surface markers by flow cytometry (Figure 6B). FACS staining showed that PECAM-1 and CD44 were expressed on vimentin-null MLEC at levels comparable to WT cells. However, ICAM-2 expression was reduced on vimentin-null MLEC compared to WT cells. We next tested the internalization of CD443MUT-A568 by MLEC. We found that WT MLEC endocytosed CD443MUT comparably to HUVEC after 30 min uptake, whereas CD443MUT uptake by MLECs isolated from vimentin-null mice was inhibited (Figure 6C).

## Discussion

We have identified vimentin as a novel CD44 binding protein. Our results – the fact that recombinant CD44HABD and CD443MUT pulled down both endogenous as well as recombinant vimentin, and the finding that vimentin displaces CD443MUT bound to HUVEC cells, suggest that CD44-vimentin interaction is a direct protein-protein interaction. To our knowledge, CD44-vimentin interaction is the first protein-protein interaction described for CD44 HABD. CD44 HABD mediates low affinity interactions with its ECM ligand HA with an *in vitro* Kd of 50 µM [23]. CD44 is a membrane glycoprotein and interacts via its glycosylated variant exons with various extracellular ligands, including fibronectin, collagen XIV, E-selectin and osteopontin [44]–[47]. CD44 HABD contains five N-linked glycosylation sites [48]. Our experiments, where glycosylated EC-endogenous or tumor cell over-expressed full-length CD44 immunoprecipitated vimentin correlate with our initial findings obtained with soluble recombinant CD44HABD or CD443MUT and strongly suggest that post-translationally modified CD44 can also form a complex with vimentin. However, we were not able to detect full-length CD44 in anti-vimentin antibody immunoprecipitates from HUVEC lysates, which can be explained by the fact that while HUVEC express high levels of vimentin, only a small fraction forms a complex with membrane bound CD44.

We found that CD44 HABD binds to vimentin within its head domain. Vimentin head-domain interactions include ankyrin binding at the plasma membrane [49], vimentin head-domain is also important in filament formation [50]. Our finding that CD44 binds to vimentin head domain is consistent with the proposed vimentin structure. Parallelly aligned dimers of vimentin assemble laterally into tetramers in a fashion whereby first halves of antiparallel coiled-coil domains overlap. Physiologically, vimentin forms a non-polar 32-meric unit-length filaments (ULF) consisting of 16 dimers or 8 tetramers [51]. The observed stoichiometries of 6–10 moles of vimentin per one mole CD443MUT probably reflects the number of head domains available on the ULF surface. The Kd values calculated from SPR data (12–37 nM) for the high affinity binding site are about 2–5 times higher than Kd-s resulting from ITC experiments (74 nM). Such experimental discrepancy can be explained either by limited dynamics of the immobilized vimentin or by sterical hindrances in the environment of the SPR chip. Currently the exact model of vimentin binding of CD44 or whether its binding site coincides with the HA binding surface, is not known. However, our data show that pharmacophores for HA-binding are not necessary for vimentin binding. Our data suggest a protein-protein interaction model which is constrained by the fact that CD44 is a type I membrane receptor and vimentin is a cytoplasmic intermediate filament protein. Nevertheless, several independent findings make this interaction spaciotemporally feasible. In addition to generation of CD44 intracellular domain resulting from shedding, full-length CD44 is also endocytosed and transported to the nucleus via NLS located in its intracellular domain [52], [53]. In this process CD44 acts as scaffold for STAT3 and p300 [53]. Importantly, leptomycin B induces CD44 nuclear accumulation, suggesting a nuclear-cytoplasmic shuttling [52]. On the other hand, cell surface vimentin is a well-known phenomenon without any known function. We show that cell surface vimentin is readily detectable in primary human endothelial cells, in addition to its previously reported presence in malignant lymphocytes, activated macrophages and platelets [34]–[36]. Vimentin provides bacterial binding sites on the surface of human brain endothelial cells [37]. Our results suggest that vimentin might provide a binding site for soluble CD44 on EC. This is supported by our result that exogenously added vimentin can efficiently displace CD443MUT from ECs. In addition, we found that CD443MUT EC binding was enhanced by VEGF. These results were confirmed by experiments of cell surface biotinylation of starved or VEGF-induced ECs showing that CD443MUT was able to pull-down biotinylated vimentin from VEGF-treated but not from serum starved ECs. The discrepancy between the binding of CD443MUT to starved EC in cellular binding experiment and lack of any detectable biotinylated vimentin in pull-downs from starved EC could be explained by the different length of serum starvation in these experiments (6 h v. over-night, respectively). We suggest that the physiological relevance of these results is supported by findings that vimentin and CD44 are up-regulated on tumor endothelial cells, whereas vimentin has been proposed as a potential anti-angiogenesis target [3], [38].

Here we show that after binding CD44HABD and its non-HA-binding triple mutant are endocytosed by ECs. A fraction of CD44HABD-proteins colocalized with generic endocytosis tracer CTxB-positive vesicles and were targeted to early endosomal structures. Importantly, we found that CD443MUT uptake was lost in vimentin knock-out endothelial cells, suggesting further that such internalization is mediated by vimentin. The number of CD443MUT-positive vesicles and early-endosomal localization decreased rapidly, most probably suggesting its targeting to lysosomal degradation. However, we were not able to detect significant accumulation of fluorescently labeled CD443MUT within late endosomal compartment.

We propose that vimentin forms a complex with full-length CD44. In this model, soluble CD44 antagonizes binding of membrane CD44 to vimentin. However, the role for soluble CD44 in tumorigenesis still remains elusive, as highly elevated soluble CD44 associates with aggressive growth and bad prognosis in cancer patients, and yet our previous results suggest that recombinant CD44 administration can inhibit tumor xenograft growth and angiogenesis [27]. We can speculate, that in cancer patients with high sCD44, tumor cells have acquired resistance to its inhibitory effects, while shedding of cell-surface bound CD44 confers significant selective advantage in tumor microenvironment. In summary, given the facts that the expression of CD44 and vimentin correlate with EMT in cancer cells, and with tumor angiogenesis, our findings provide rationale for further functional studies on the role of these proteins in EMT and angiogenesis.

## Materials and Methods

### Cell lines and antibodies

HUVEC and MLEC cells were grown in M199 medium supplemented with 20% FBS, 4 mM L-glutamine, 50 µg/ml heparin and 30 µg/ml EC growth supplement (ECGS, Upstate Biotechnology, Lake Placid, NY, USA). MCF-7 cells (ATCC, Manassas, VA, USA) were grown in RPMI, supplemented with 10% FBS and 2 mM L-glutamine. Anti-vimentin (V9), anti-Myc (A-14) and anti-HDAC1 (H-11) antibodies were from Santa Cruz Biotechnology (Santa Cruz, CA, USA). Anti-vimentin rabbit polyclonal (18-272-196311) was from Genway (San Diego, CA, USA). Anti human-CD44 (2C5) was from R&D Systems (Minneapolis, MN, USA). Mouse anti-human CD44 (H4C4) was from DSHB (University of Iowa, IA, USA). Anti CD443MUT mouse mAb 1A2 (Figure S2) was generated by LabAS Ltd (Tartu, Estonia). Anti-CD44 (MEM-263) was from EXBIO Praha (Czech Republic). Anti-mouse PECAM-1 (MEC13.3), anti-mouse ICAM-2 (3C4) and anti-EEA1 mAb were from BD Pharmingen (Palo Alto, CA, USA). Rat anti-CD63/lamp-3 (R5G2) was from MBL International (Woburn, MA, USA). Anti-Flag-M2 antibody was from Sigma.

### Purification of recombinant proteins and fluorescence labeling

CD44HABD and CD443MUT GST fusion-proteins were purified as described [27]. CD44HABD and CD443MUT include aa 21–132 of human CD44 protein. CD44HABD and -3MUT were expressed using pET11c vector (Novagen). Urea dissolved inclusion bodies were purified by gel filtration in Superdex-200HR 16/60 column (GE Healthcare, Uppsala, Sweden). Refolding was performed by gradient dialysis into 50 mM Tris pH 8.0, 150 mM NaCl and final dialysis into PBS. Endotoxin level was measured using the Endosafe-PTS (Charles River, L'Arbresle, France). Endotoxin values of CD443MUT batches were 22–93 EU/mg. Human vimentin was expressed using pET15b vector (Novagen). His-tagged vimentin was purified using Ni-affinity resin (Sigma) under denaturing conditions. Refolding was performed by gradient dialysis into 10 mM Tris pH 8.0 with final dialysis into 10 mM phosphate buffer pH 7.4. Proteins were fluorescence-labeled using sulfo-NHS-Alexa Fluor 488 or −568 protein labeling kit (Molecular Probes, Eugene, OR, USA).

### GST pull-down, immunoprecipitation and cell surface biotinylation

Adherent cells were rinsed with ice-cold PBS and lysed on ice in 50 mM Tris pH 8.0, supplemented with protease inhibitor cocktail (PIC; Roche, Mannheim, Germany). Lysate was centrifuged at 14000 rpm for 30 min at 4°C. Pellet was solubilized in 2% CHAPS, 50 mM Tris pH 8.0, 50 mM NaCl, PIC buffer and centrifuged at 14000 rpm for 10 min at 4°C. Supernatant was precleared by incubation with GST-bound glutathione-sepharose 4FF beads (Amersham Biosciences, Uppsala, Sweden). Precleared lysate was incubated overnight at 4°C with 10 µg GST, GST-tagged CD44HABD or CD443MUT immobilized onto glutathione beads. After washes with 50 mM Tris pH 8.0, 150 mM NaCl, PIC buffer, beads were eluted with 20 mM reduced glutathione in 50 mM Tris pH 8.0. Eluates were precipitated with 20% TCA, precipitate was washed with cold acetone and aspirated dry. For MALDI-TOF MS analysis of tryptic peptides, protein samples were alkylated and visualized by silver staining on SDS-PAGE.

For biotinylation, adherent cells were incubated with 1 mM EZ-Link Sulfo-NHS-LC-biotin (Pierce, Rockford, IL, USA) in PBS-0.05% NaN_3_ for 30 min on ice, washed with 100 mM glycine-PBS and lysed as described above. For IP of endogenous proteins, adherent cells were rinsed with cold PBS and lysed in 50 mM Tris pH 8.0, 50 mM NaCl, 1% CHAPS, PIC buffer. Lysate was centrifuged at 14000 rpm for 30 min at 4°C. Supernatant was pre-cleared with anti-HDAC1 immobilized onto protein A/G sepharose beads (Amersham Biosciences) at 4°C. Precleared lysate was incubated with anti-HDAC1 or anti-CD44 (MEM-263) antibodies immobilized onto protein A/G beads overnight at 4°C. Beads were washed with lysis buffer and bound proteins were eluted with 0.5 M glycine (pH 2.5). Finally, pH of eluates was adjusted with 1 M Tris pH 8.0 and they were analyzed by immunoblotting using anti-CD44 (2C5) or rabbit anti-vimentin antibody. For IP of over-expressed proteins, adherent cells were rinsed in cold PBS, lysed in lysis buffer containing 40 mM Hepes pH 7.4, 120 mM NaCl, 1 mM EDTA, 0.6% CHAPS and PIC. Lysates were centrifuged at 14000 rpm for 30 min at 4°C. Supernatants were incubated with anti-Flag-M2 affinity gel (Sigma) overnight at 4°C, beads were washed with lysis buffer and bound proteins were eluted with 2× Laemmli sample buffer. Eluted protein complexes were analyzed by immunoblotting with anti-Flag-M2 or anti-Myc.

### Isothermal titration calorimetry and surface plasmon resonance

ITC measurements were performed on a Nano-2G instrument (TA Instruments, New Castle, DE, USA). Experiments were performed in 50 mM Tris, 150 mM NaCl, pH 8.0 at 25°C. The main experimental parameters were: sample cell volume – 1 ml, syringe size – 250 µl, stirring rate – 250 rpm, injection volume – 10 µl, time interval between injections – 300 s. Titration data were analyzed by non-linear fitting (SigmaPlot 10). SPR measurements were performed on Biacore3000 (GE Healthcare). Vimentin was covalently coupled to CM5 chip using amine coupling kit (GE Healthcare). In association phase, CD443MUT concentrations 0.46–123 µM were injected over the chip surface. In the dissociation phase, the sensor chip surface was eluted with buffer 50 mM Tris, 150 mM NaCl, pH 8.0. The association rate constants and the dissociation rate constants were estimated using BIAevaluation software (GE Healthcare) using a parallel binding model, A+B1 ↔AB1, A+B2 ↔ AB2. K_d_ values were also determined from analysis of the equilibrium data using equation 1: ΔR = (ΔR_max_·x)/(K_d_+x)+(c · x), where x – concentration of the injected protein, ΔR – the increase of the response value at equilibrium, ΔR_max_ – capacity of the immobilised vimentin to bind a protein (the number of binding sites), and c describes weak or non-specific interaction.

### Displacement assays

Adherent cells were harvested from culture plates with 5 mM EDTA in PBS. Proteins were iodinated with ^125^I by using Iodo-beads (Pierce). Cells were resuspended in incubation buffer 20 mM Tris-HCl pH 7.5, 5 mM MgCl_2_, 30 mM NaCl, 3 mM CaCl_2_ or DMEM, 25 mM HEPES, 0.1% BSA. Cell suspension was transferred into 96-well microtitre plate in 100 µl volume. Unlabeled protein at different concentrations and ^125^I labeled protein in 20 µl volume of incubation buffer was added into wells. Reactions were incubated overnight at 4°C and stopped by filtration through GF/B filters blocked with 0.1% BSA-PBS, followed by washes with cold PBS. Filters were transfered into 5 ml vials and bound radioactivity was measured using gamma counter (PerkinElmer).

### FACS analyses

For CD443MUT cellular binding, HUVEC were serum starved 6 h and then induced for 30 min at 37°C with 10 ng/ml VEGF-165 in media containing 0.5% FBS. Alexa Fluor 488-conjugated CD443MUT or GST was added into media at 25 µg/ml and cells were incubated for 1 h on ice. Cells were harvested from culture plates by scraping. After washes with 0.1% BSA-PBS, cells were fixed in 4% formaldehyde-PBS and analyzed using FACSCalibur flow cytometer (BD Biosciences).

### DNA constructs and transfection

Full-length vimentin was PCR amplified from human vimentin cDNA and inserted into EcoRI/SacII site of pcDNA3.1/MycHisB vector (Invitrogen). Vimentin deletion mutants containing amino acids 1-96 (VIM1-96), 1-245 (VIM1-245), 246-466 (VIM246-466) and 97-466 (VIM97-466) were PCR amplified from human vimentin cDNA using oligonucleotide pairs containing EcoRI/NotI sites. PCR fragments were inserted into EcoRI/NotI site of pcDNA3.1/MycHisB vector. Vimentin-GFP (GFP, green fluorescent protein) constructs were created by inserting EcoRI/SacII fragment from respective vimentin-pcDNA3.1/MycHisB constructs into pEGFP-N1 vector. Vimentin deletion mutant containing aa 407-466 (VIM407-466) was PCR amplified from human vimentin cDNA and inserted into EcoRI/SalI site of pEGFP-C2 vector. For creating Flag-tagged CD44 DNA construct, full-length CD44 was PCR amplified from human standard CD44 isoform cDNA and inserted into EcoRI/NotI site of pCMV-Tag4a vector (Stratagene). MCF-7 cells were transfected using 1∶2 DNA∶PEI ratio. Transfected cells were grown at 37°C for 24 h. GST pull-down was performed as described above.

### Mouse lung endothelial cells

Wild-type MLEC were isolated from C3H mouse strain (The Jackson Laboratory) and vimentin−/− from Vim1/Vim1 mice [31] obtained from EMMA (CNRS/CDTA, Orleans, France). Lungs from three 6–8 week old mice were dissected and finely minced with scissors on a dry culture dish. Lung pieces were put into 20 ml pre-warmed 0.2% collagenase-I (Sigma) in PBS and incubated with gentle agitation for 45 min at 37°C. Collagenase digested lung suspension was triturated through 100 µm cell strainer (BD Biosciences). Cell suspension was centrifuged 8 min 400 *g* at 4°C. Cell pellet was resuspended in 2 ml 0.1% BSA-PBS. Cells were sorted by incubation for 15 min at RT with sheep anti-rat IgG Dynabeads (Dynal, Norway) coated with rat anti-mouse CD31 (MEC13.3) and rat anti-mouse ICAM-2 (3C4) antibodies. Bead-bound cells were separated using a magnetic rack and washed five times with M199 medium containing 10% FBS. After separation, cells were plated onto dish and grown in M199 containing 10 mM HEPES, 20% FBS, 4 mM L-glutamine and supplemented with 50 µg/ml Heparin, 30 µg/ml ECGS and penicillin-streptomycin.

### Internalization assay, immunofluorescence microscopy and image processing

For internalization assays, cells on 8-well slide (BD Falcon) were incubated at 37°C with CTxB-Alexa 555 (Invitrogen) and/or CD44HABD-proteins at 13 µg/ml (≈1 µM) in 0.5% FBS containing media for 10 or 30 min. After 10 min uptake, cells were washed with PBS two to three times and media was changed to 10% FBS containing M199 HUVEC growth media and slides were incubated for 20 or 50 min at 37°C. After incubations cells were washed and fixed with 4% formaldehyde-PBS on for 10 min on ice and for 10 min at RT. Cells were permeabilized using 0.1% Triton X-100 in 0.1% BSA-PBS. Antibodies were diluted in 0.1% BSA-PBS. Secondary antibody dilutions were supplemented with 10 µg/ml Hoechst 33258 (Sigma). Slides were mounted in Mowiol 4–88 (Sigma-Aldrich, St Louis, MO, USA). Confocal fluorescent imaging was performed using Zeiss LSM510 microscope with ×63/1.4 oil immersion objective in multi-channel mode (Carl Zeiss MicroImaging, Germany). Images were prepared using Imaris 6.4 software (Bitplane, Zurich, Switzerland). For quantitation of endocytosis and vesicular colocalization, single slices from the middle plane of the cell were semi-automatically selected from confocal image stacks using Fiji package (http://pacific.mpi-cbg.de/wiki/index.php/Fiji). Cellprofiler 2.0 (r10415) software was used ror image segmentation and automated analysis [54]. Endosomal outlines were identified using Otsu global treshold, then endosomal marker/tracer object outlines were used to create a mask to identify colocalizing CD44HABD- or CD443MUT objects. Within these objects correlation was measured between endocytosis marker and CD44, and objects showing positive correlation were finally counted as colocalizing. For calculation of average correlation coefficient and 95% confidence interval, individual object coefficients were transformed to z scores.

### Statistical analysis of data

Data represent mean ± SE. Statistical analysis and non-linear fitting of data was performed using GraphPad Prism 5 software (San Diego, CA, USA).

## Supporting Information

Figure S1 **Cell-surface expression of overexpressed vimentin in MCF-7 cells.** Vimentin- or empty vector transfected MCF-7 cells were subjected to cell surface biotinylation (see Materials and Methods). Lysates were immunoprecipitated with anti-vimentin antibody. Lysates and immunoprecipitates were analyzed by WB using strepavidin-HRP (upper panel) or anti-vimentin antibody (lower panel). Arrows indicate the location of full length vimentin.
(TIF)

Figure S2 **Characterization of anti-CD443MUT mouse mAb 1A2.** (A) ELISA analysis of serially diluted 1A2 mAb (3.1 mg/ml) of rat serum−, rat serum+CD443MUT- or CD443MUT-coated wells. PBS, no primary antibody control. (B) Microplate wells were coated with different concentrations of CD443MUT mixed with rat serum and analyzed by ELISA using 1A2 mAb at 1∶400 dilution. (C) Wells were coated with CD44 peptides and analyzed by ELISA using 1A2 mAb at 1∶50000 dilution. (D) Amino acid alignment of CD44HABD, CD443MUT and peptides used for epitope mapping. Amino acid numbering is according to human CD44; mutated positions are indicated in green (wild-type amino acids) or red (mutant amino acids). Bars, mean ± SD.
(TIF)
