# Supplementary figures and images for: Soluble CD44 Interacts with Intermediate Filament Protein Vimentin on Endothelial Cell Surface
Source: PLoS One. 2011 Dec 21;6(12):e29305. doi: 10.1371/journal.pone.0029305 (PMC3244446; doi:10.1371/journal.pone.0029305)

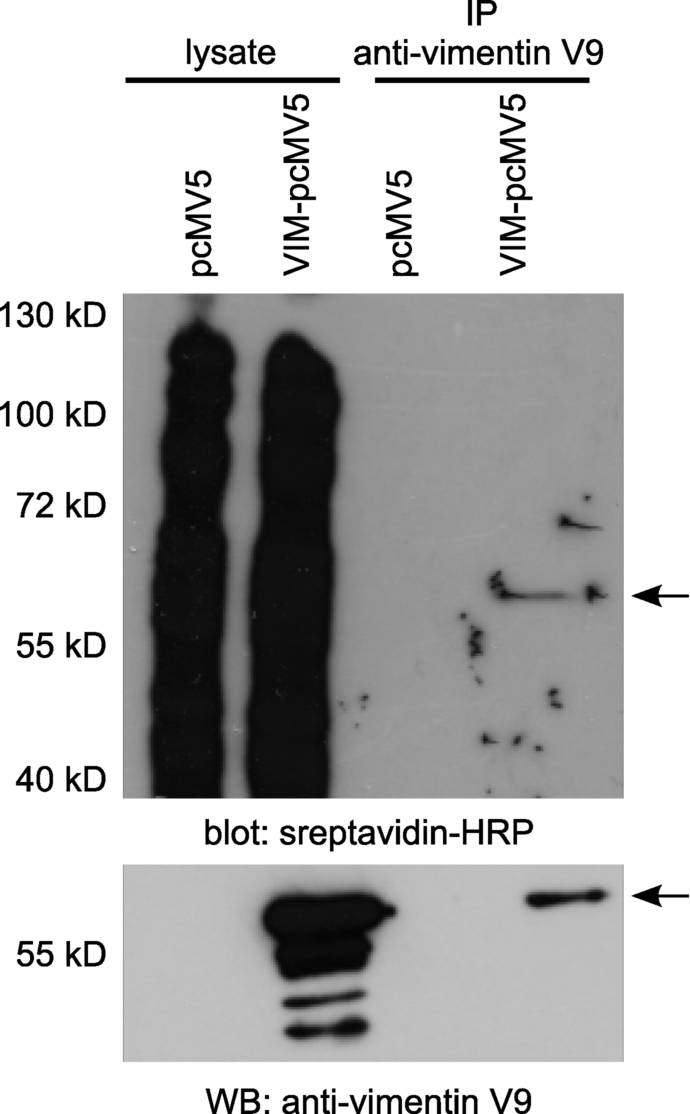

Supplement: Figure S1 — Cell-surface expression of overexpressed vimentin in MCF-7 cells. Vimentin- or empty vector transfected MCF-7 cells were subjected to cell surface biotinylation (see Materials and Methods). Lysates were immunoprecipitated with anti-vimentin antibody. Lysates and immunoprecipitates were analyzed by WB using strepavidin-HRP (upper panel) or anti-vimentin antibody (lower panel). Arrows indicate the location of full length vimentin. (TIF) [file pone.0029305.s001.tif]

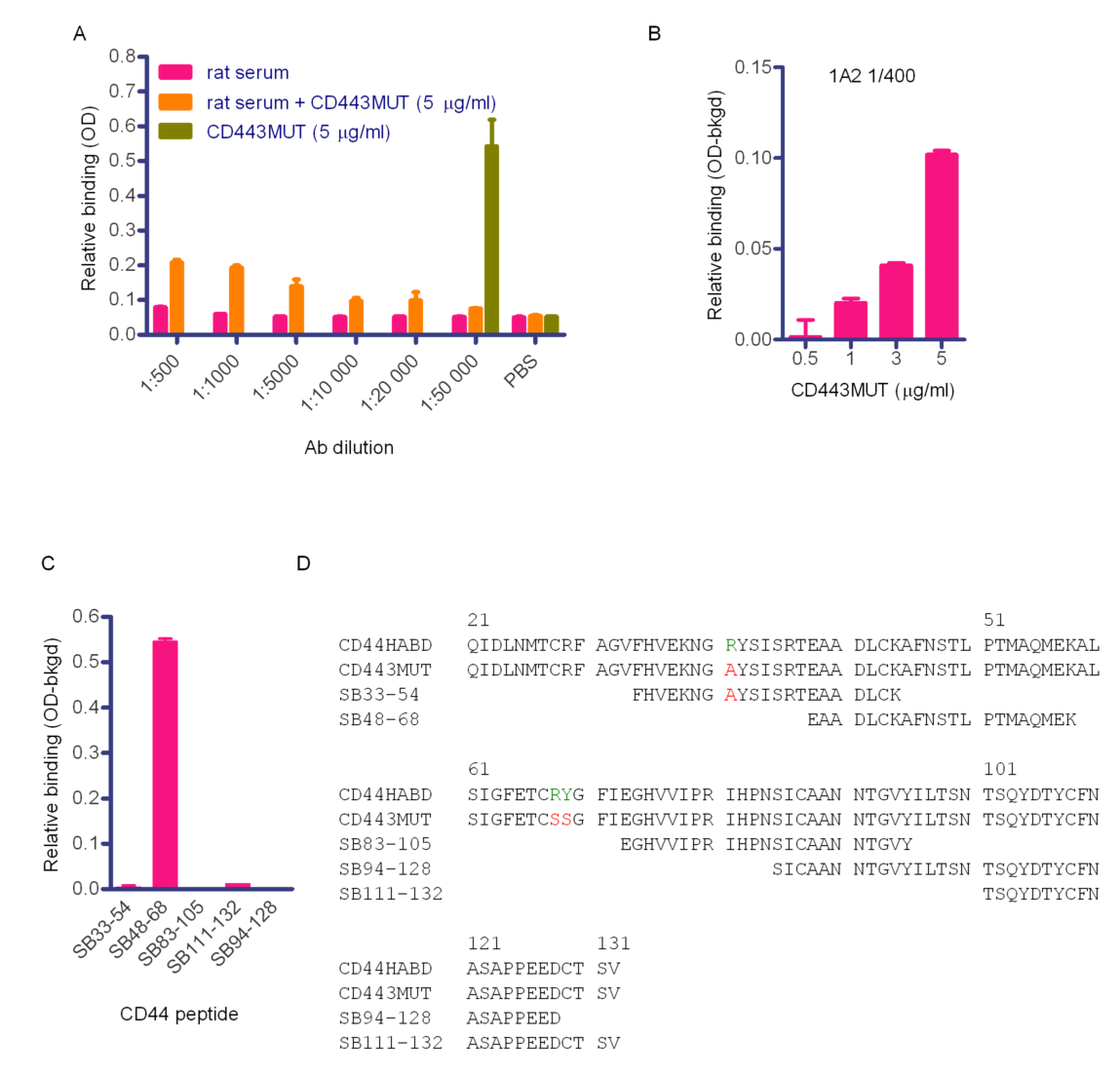

Supplement: Figure S2 — Characterization of anti-CD443MUT mouse mAb 1A2. (A) ELISA analysis of serially diluted 1A2 mAb (3.1 mg/ml) of rat serum−, rat serum+CD443MUT- or CD443MUT-coated wells. PBS, no primary antibody control. (B) Microplate wells were coated with different concentrations of CD443MUT mixed with rat serum and analyzed by ELISA using 1A2 mAb at 1∶400 dilution. (C) Wells were coated with CD44 peptides and analyzed by ELISA using 1A2 mAb at 1∶50000 dilution. (D) Amino acid alignment of CD44HABD, CD443MUT and peptides used for epitope mapping. Amino acid numbering is according to human CD44; mutated positions are indicated in green (wild-type amino acids) or red (mutant amino acids). Bars, mean ± SD. (TIF) [file pone.0029305.s002.tif]
